# Real-Time Neurofeedback to Modulate β-Band Power in the Subthalamic Nucleus in Parkinson’s Disease Patients

**DOI:** 10.1523/ENEURO.0246-18.2018

**Published:** 2018-12-21

**Authors:** Ryohei Fukuma, Takufumi Yanagisawa, Masataka Tanaka, Fumiaki Yoshida, Koichi Hosomi, Satoru Oshino, Naoki Tani, Haruhiko Kishima

**Affiliations:** 1 Department of Neurosurgery, Graduate School of Medicine, Osaka University, Suita, Osaka, 565-0871, Japan; 2 Department of Neuroinformatics, ATR Computational Neuroscience Laboratories, Seika-cho, Kyoto, 619-0288, Japan; 3 Institute for Advanced Co-creation Studies, Osaka University, Suita, Osaka, 565-0871, Japan; 4 Center for Information and Neural Networks (CiNet), National Institute of Information and Communications Technology (NICT), Suita, Osaka, 565-0871, Japan; 5 JST PRESTO, Suita, Osaka, 565-0871, Japan; 6 Department of Neuromodulation and Neurosurgery, Graduate School of Medicine, Osaka University, Suita, Osaka, 565-0871, Japan

**Keywords:** beta power, deep brain stimulation, EEG, neurofeedback, Parkinson disease, voluntary control

## Abstract

The β-band oscillation in the subthalamic nucleus (STN) is a therapeutic target for Parkinson’s disease. Previous studies demonstrated that l-DOPA decreases the β-band (13–30 Hz) oscillations with improvement of motor symptoms. However, it has not been elucidated whether patients with Parkinson’s disease are able to control the β-band oscillation voluntarily. Here, we hypothesized that neurofeedback training to control the β-band power in the STN induces plastic changes in the STN of individuals with Parkinson’s disease. We recorded the signals from STN deep-brain stimulation electrodes during operations to replace implantable pulse generators in eight human patients (3 male) with bilateral electrodes. Four patients were induced to decrease the β-band power during the feedback training (down-training condition), whereas the other patients were induced to increase (up-training condition). All patients were blinded to their assigned condition. Adjacent contacts that showed the highest β-band power were selected for the feedback. During the 10 min training, patients were shown a circle whose diameter was controlled by the β-band power of the selected contacts. Powers in the β-band during 5 min resting sessions recorded before and after the feedback were compared. In the down-training condition, the β-band power of the selected contacts decreased significantly after feedback in all four patients (*p* < 0.05). In contrast, the β-band power significantly increased after feedback in two of four patients in the up-training condition. Overall, the patients could voluntarily control the β-band power in STN in the instructed direction (*p* < 0.05) through neurofeedback.

## Significance Statement

Many studies have reported a relationship between the β-band power in the subthalamic nucleus (STN) and motor symptoms in Parkinson’s disease. Here, we have developed a novel neurofeedback technique using intracranial electrodes implanted in deep brain structures to modulate STN activity. We provided direct feedback of the β-band power as the size of a black disk to induce a sustainable change in β-band power. As a result, the neurofeedback training induced significant changes in the β-band power. This is the first report to demonstrate that human patients with Parkinson’s disease were able to voluntarily control their β-band power in STN to induce changes in the power.

## Introduction

Parkinson’s disease is characterized by abnormal neuronal oscillations in the subthalamic nucleus (STN). Electrophysiological examinations using electrodes for deep-brain stimulation (DBS) have demonstrated that the β-band oscillations in the STN correlate with the symptoms of Parkinson’s disease (Little and Brown, 2012; Pavlides et al., 2015). In addition, treatment with dopaminergic (l-DOPA) medication improves Parkinson’s disease symptoms, such as bradykinesia and rigidity, while simultaneously attenuating β-band power (Brown et al., 2001; Cassidy et al., 2002; Priori et al., 2004; Kühn et al., 2006a; Weinberger et al., 2006; Hammond et al., 2007; Ray et al., 2008). Similarly, DBS in the STN suppresses β-band oscillation (Eusebio et al., 2011). Moreover, recent studies have demonstrated that an adaptive DBS using β-band oscillation improved Parkinson’s disease symptoms better than the continuous use of DBS. These improvements were correlated with the attenuation of β-band oscillations (Little et al., 2013; Tinkhauser et al., 2017), so β-band oscillation in the STN may be a therapeutic target for clinical interventions such as rehabilitation.

However, it has not been revealed whether patients with Parkinson’s disease voluntarily modulate the β-band oscillation in the STN for rehabilitation. Because the β-band oscillation in the STN is a part of the cortico–basal ganglia–thalamocortical network, it is affected by various voluntary activities such as motor intentions (Blumenfeld and Brontë-Stewart, 2015). Previous studies have demonstrated coherent oscillations, including β-band throughout the network, such as STN and internal globus pallidus (GPi; Brown et al., 2001), GPi and cortex (Williams et al., 2002), STN and thalamus (Hanson et al., 2012), and STN and cortex (Litvak et al., 2011; Whitmer et al., 2012; de Hemptinne et al., 2013). It has also been reported that not only actual hand movement but also mental imagery to move the hand changes the β-band power in the STN of patients with Parkinson’s disease (Kühn et al., 2006b), which is affected by the cortical activations linked to the basal ganglia (Raffin et al., 2012; Blumenfeld and Brontë-Stewart, 2015). Voluntary modulation of β-band oscillation in the STN might, therefore, induce some plastic changes in activities.

Neurofeedback has been demonstrated to induce plastic changes in various cortical activities (Emmert et al., 2016), including those in Parkinson’s disease (Beuter et al., 2014). Studies using real-time monitoring of cortical activities demonstrated that neurofeedback could induce changes in cortical activity and function (Ganguly et al., 2011; Wander et al., 2013; Orsborn et al., 2014). For some patients after strokes, neurofeedback with magnetoencephalography and electroencephalography successfully modulated the α or β power of the cortical current such that the patients’ symptoms improved (Buch et al., 2008; Ramos-Murguialday et al., 2013; Chaudhary et al., 2015). Hence, the β-band oscillation in the STN of patients with Parkinson’s disease might be modulated through the neurofeedback training. Here, we hypothesized that patients with Parkinson’s disease could control the intensity of the β-band oscillation of the STN using real-time feedback of the STN recordings. Moreover, the motor symptoms of the patients were evaluated by electromyograms (EMGs) of their upper limbs to examine the relationship with the β-band oscillation of the STN.

## Materials and Methods

### Patients

Eight patients with bilateral STN-DBS electrodes (3 males and 5 females) were recruited in the Neurosurgery Department of Osaka University Hospital at a location which will be identified if the article is published (Table 1; for DBS parameter settings, see Table 2). The ethics committee of Osaka University Hospital approved this study (no. 14448), and it was performed in accordance with approved protocols. All patients gave written informed consent to participate before the experiment.

**Table 1:** Patients and feedback conditions

**Patient ID**	**Age, y (sex)**	**Duration of DBS, y**	**UPDRS-III (On)**	**Feedback condition**	
**Contacts**	**Group**				
1	53 (M)	11	27	Lt 1–2	Down-training
2	70 (M)	4	29	Lt 1–2	Down-training
3	68 (F)	6	7	Lt 1–2	Down-training
4	52 (F)	5	20	Lt 0–1	Down-training
5	62 (F)	4	26	Lt 0–1	Up-training
6	66 (M)	9	27	Rt 1–2	Up-training
7	67 (F)	9	82	Rt 1–2	Up-training
8	66 (F)	4	31	Rt 1–2	Up-training

UPDRS-III, Unified Parkinson’s Disease Rating Scale Part III; Rt, right; Lt, left.

**Table 2.** DBS parameter settings

**Patient ID**	**Contacts**	**Frequency, Hz**	**Pulse width, μs**	**Voltage**
1	Lt 1 − 2− C+	130	60	3.4
Rt 2 − 3− C+	60	3.5		
2	Lt 2 − 3− C+	130	90	3.0
Rt 2 − 3− C+	90	2.4		
3	Lt 2− C+	60	60	3.9
Rt 0− C+	90	3.8		
4	Lt 0 − 1− C+	60	90	3.2
Rt 0− C+	90	3.2		
5	Lt 1− C+	60	90	4.1
Rt 1− C+	90	4.1		
6	Lt 2 − 3− C+	125	60	2.7
Rt 2− C+	90	2.6		
7	Lt 2− C+	60	90	3.9
Rt 2− C+	90	4.0		
8	Lt 2 − 3− C+	140	60	3.2
Rt 1 − 2− 3− C+	130	90	2.8	

Rt, Right; Lt, left.

### Signal measurement

During operations with local anesthesia for replacement of implantable pulse generators, signals from bilateral DBS electrodes were measured at 10 kHz by electroencephalograph (EEG; NIHON KOHDEN). The DBS electrode was 1.27 mm in diameter and had four contacts on its tip in the axial direction (Model 3389, Medtronic). Each contact was 1.5 mm long, and the spacing between contacts was 0.5 mm. EMG from the flexor digitorum superficialis and the extensor digitorum communis of each hand were also measured at the same time to evaluate symptoms. These muscles were selected as the antagonistic muscle pairs that were accessible even during the operation.

### Experimental design

**Figure 1.**
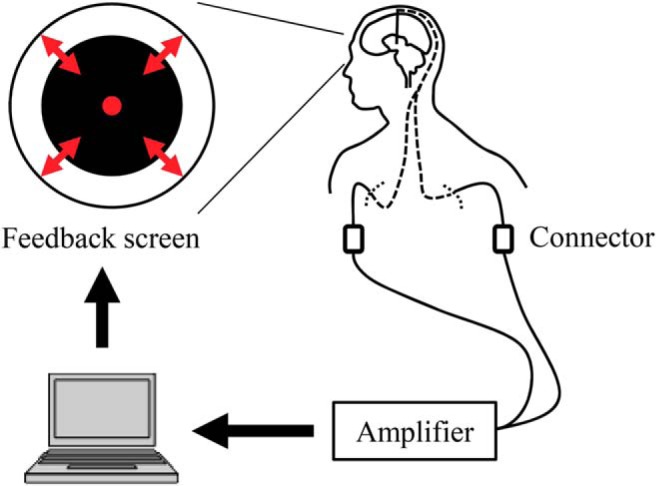
Feedback system overview. Signals from the DBS electrodes were acquired in real time. The radius of the black circle on the computer screen was controlled based on the β-band power of the acquired bipolar signals from adjacent contacts that were selected in the pre-feedback session.

The experiment was performed with patients lying on the surgical bed and 2–3 h after medication was administered. Each patient participated in three sessions in the following order: pre-feedback session, feedback session, and post-feedback session. In the 5 min pre- and post-feedback sessions, the patients were instructed to close their eyes and not to fall asleep. During the 10 min feedback session, patients were instructed to make the radius of a black circle on a computer screen smaller by using their thoughts somehow, without moving their bodies (Fig. 1). The computer screen was fixed in front of the patient’s face, ∼20–40 cm away, so that the patient could comfortably see the black circle, which had a maximum radius of ∼10 cm. Movements of the body were visually monitored; in addition, those of the hands were also monitored using EMG. The radius of the circle was controlled by β-band power scaled in the range of 0–1, in two directions (for the scaling method, see Real-time feedback). For four patients in the down-training group, the radius was proportional to the normalized power so that the scaled power of 0 showed no black circle, and that of 1 gave the maximum radius of the circle. In contrast, for the other four patients in the up-training group, the radius was negatively correlated to the scaled power to give the maximum radius with the value of 0.

### Real-time feedback

During the pre-feedback and feedback sessions, β-band power was calculated in real-time using a script running on MATLAB (MathWorks). Measured signals were first transferred from EEG to MATLAB via TCP/IP. At 50 ms intervals, the last 500 ms bipolar signals from adjacent contacts were applied with a Hamming window and fast Fourier transformation to obtain the power spectrum. The power spectrum within the β-band was averaged, and the square root was calculated to find the β-band power. In this series of procedures, only functions built into MATLAB, or supplied in MathWorks toolboxes were used to calculate power. Adjacent contacts that showed the highest β-band (13–30 Hz) power during the pre-feedback session were selected for the contacts to control the circle during the subsequent feedback session. During the feedback session, the β-band power of the selected adjacent contacts, calculated in real-time, was scaled into a range of 0–1 to control the radius of the feedback circle. The scaling was performed so that lower limit (0) and upper limit (1) of the range corresponded to the minimum and maximum power, respectively, of the same contacts during the pre-feedback session. If the scaled power exceeded the range of 0–1, the scaled power was clipped within the range so that the maximum and minimum radius of the circle was limited. The radius of the feedback circle was sent via serial port to another computer, on which the feedback circle was displayed using in-house custom software.

### Signal processing

To evaluate the changes induced by the feedback training, the β-band power of the 10 kHz sampled DBS signals was calculated from the signals recorded during the pre- and post-feedback sessions. At first, noisy portions of the recordings were discarded based on visual inspection before further analysis, and the clean signals were divided into non-overlapping 1 s time windows. For each time window, the DBS signals from the selected contacts for the feedback training were applied with a Hamming window and fast Fourier transformation to obtain a power spectrum. The β-band power of each time window was obtained as the square root of the averaged spectrum between 13 and 30 Hz.

The power of the EMG signals measured from the forearm contralateral to the selected DBS contacts was also calculated to evaluate the effect of feedback training on the symptoms. The EMG signals were processed in the same manner as the DBS signals, except the power spectra were averaged between 4 and 10 Hz from the flexor digitorum superficialis and the extensor digitorum communis to calculate the EMG power.

The β-band power of the DBS signals from the selected contacts, and the EMG power from the contralateral forearm were also calculated using the recording during the feedback training. Calculations of both powers were performed in the same manner as in that of the rest sessions, except the signals during the feedback task were divided into 600 non-overlapping 1 s time windows.

### Statistics

The β-band power of the selected DBS contacts was compared between the pre- and post-feedback sessions to evaluate the effect of feedback training. For each patient, the β-band powers of the 1 s time windows during the two rest sessions were compared with a one-tailed unpaired *t* test to evaluate whether each patient successfully induced changes in the β-band powers in the instructed direction. Moreover, to test whether the patients could control the β-band power according to the instructions as a group, the difference of the averaged β-band power during the two rest sessions was evaluated. For the down-training group, the difference was calculated as the power of pre-feedback session subtracted from that of post-feedback session (post − pre); for the up-training group, the power of the post-feedback session was subtracted from that of pre-feedback session (pre − post). By applying one-sample *t* test to the differences, the *t* value was calculated; a one-tailed permutation test was performed to examine the significance of the *t* value by comparing it with a distribution of the *t* values expected by chance. The chance distribution was obtained by randomly shuffling the powers of the two rest sessions for the same patient before taking their average, 10,000 times. The effect of the feedback training on the symptoms was also evaluated in the same manner as the β-band power using the EMG powers, which were calculated from the EMG signals of the flexor digitorum superficialis and the extensor digitorum communis, and within the frequency range of 4–10 Hz.

The relationship between the EMG power and the β-band power during feedback training was evaluated using Pearson’s correlation coefficient. For each patient, the correlation coefficient between the EMG and β-band power was calculated. Correlation coefficients expected by chance were also calculated by randomly shuffling the order of the power within each patient. The true and chance correlation coefficients were Fisher *z*-transformed and tested using a two-tailed unpaired *t* test.

## Results

**Figure 2.**
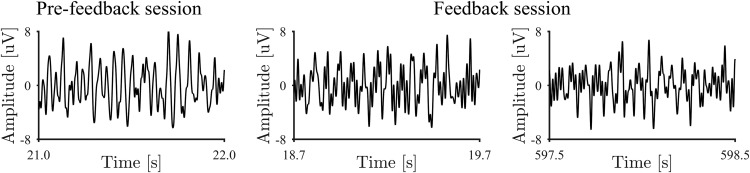
Representative DBS signals. DBS signals of Patient 2 during pre-feedback session, and at the beginning and the ending of feedback session were shown. For higher readability, the signals were bandpass filtered between 4 and 80 Hz.

**Figure 3.**
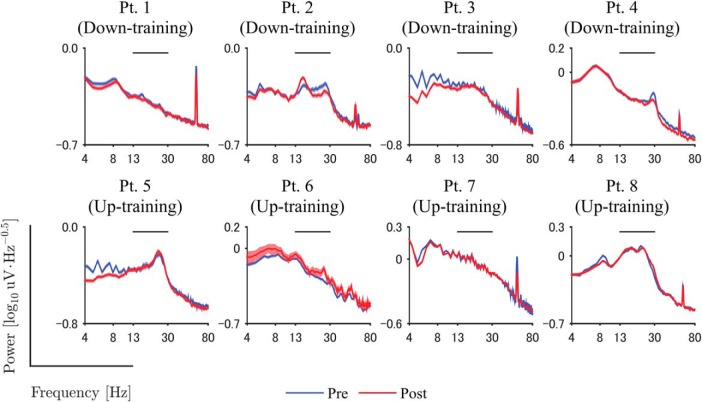
Power spectra during pre- and post-feedback sessions. Blue and red lines denote the power spectrum of DBS signals during resting state before and after the feedback training, respectively. Shaded areas represent the estimated 95% confidence interval of the power spectrum among 1 s time windows. The horizontal line above the data curves shows the range of β-band used for feedback training. Frequency is shown on a log scale.

The signals from the DBS electrodes implanted in the patients demonstrated characteristic β-band signals during the resting state. Figure 2 shows an example of the signals before the feedback training, and β-band oscillation was shown in the example. The power spectra of the DBS electrodes were evaluated during the resting states before and after the feedback training. After the first recording of the resting state, we selected the pair of adjacent DBS contacts showing the greatest β-band power during the resting state for each patient (Table 1). The power spectra from these contacts showed peaks around the β band (13–30 Hz), as shown in Figure 3.

**Figure 4.**
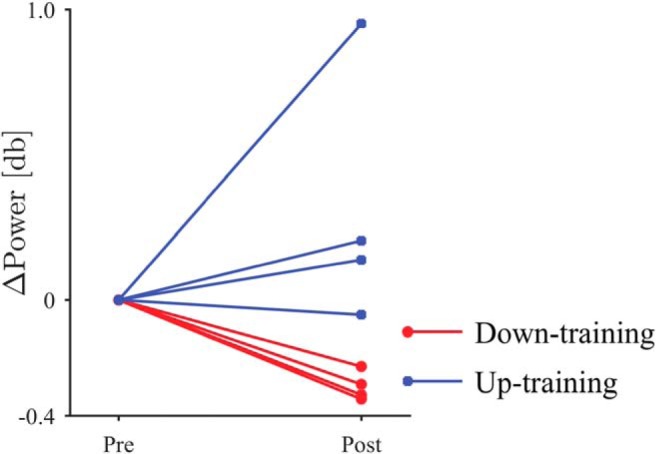
The difference in β-band power between the pre- and post-feedback sessions. The circular markers and red lines denote the down-training condition, whereas the square markers and blue lines indicate the up-training condition.

**Figure 5.**
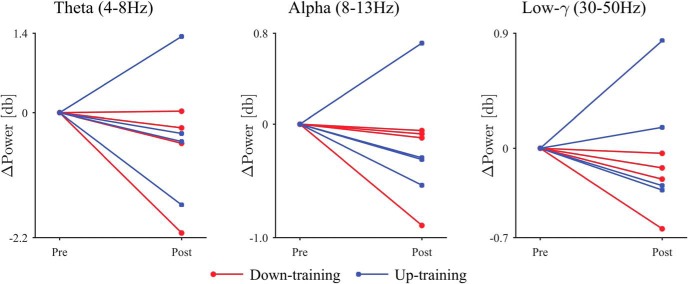
Comparison of powers between the pre- and post-feedback sessions. In common frequency bands other than β-band, difference of powers between two sessions was shown. The circular markers and red lines denote the down-training condition, whereas the square markers and blue lines indicate the up-training condition.

The neurofeedback training induced changes in the β-band power of the selected DBS contacts. The representative example of the signals demonstrated that the characteristic frequency and the amplitude changed during the neurofeedback training (Fig. 2). Figure 3 shows that the β-band power of the selected DBS contacts changed after the feedback training. For all patient except Patients 5 and 7, the β-band power was significantly changed in the targeted direction after the 10 min feedback, during which the radius of the black circle was controlled in proportion or in inverse proportion to the normalized β-band power in the STN evaluated online (*p* < 0.05, one-tailed unpaired *t* test; Table 3, a). Notably, for all patients in down-training group, the β-band power was significantly decreased after the training, whereas only two of four patients in the up-training group showed a significant increase in the β-band power. On the whole, the β-band power was significantly changed in the targeted directions after the feedback training (Fig. 4; *p* = 0.009, one-tailed permutation test; Table 3, b). The powers in other frequency bands (such as θ, α, low γ) did not, however, change significantly before and after the neurofeedback training (Fig. 5).

**Table 3.** Statistical table

	**Data structure**	**Type of test**	**Statistics**
a	Normal distribution	One-tailed unpaired *t* test	Patient 1: *t*_(598)_ = 3.286, *p* < 0.001 Patient 2: *t*_(598)_ = 2.762, *p* = 0.003 Patient 3: *t*_(598)_ = 3.013, *p* = 0.001 Patient 4: *t*_(598)_ = 4.644, *p* < 0.001 Patient 5: *t*_(598)_ = −1.241, *p* = 0.108 Patient 6: *t*_(338)_ = −3.852, *p* < 0.001 Patient 7: *t*_(598)_ = 0.743, *p* = 0.771 Patient 8: *t*_(598)_ = −1.763, *p* = 0.039
b	No assumption	One-tailed permutation test	*p* = 0.009
c	No assumption	Two-tailed permutation test	*p* = 0.627
d	Approximate normal distribution	Two-tailed unpaired *t*-test	*t*_(14)_ = 0.749, *p* = 0.466

**Figure 6.**
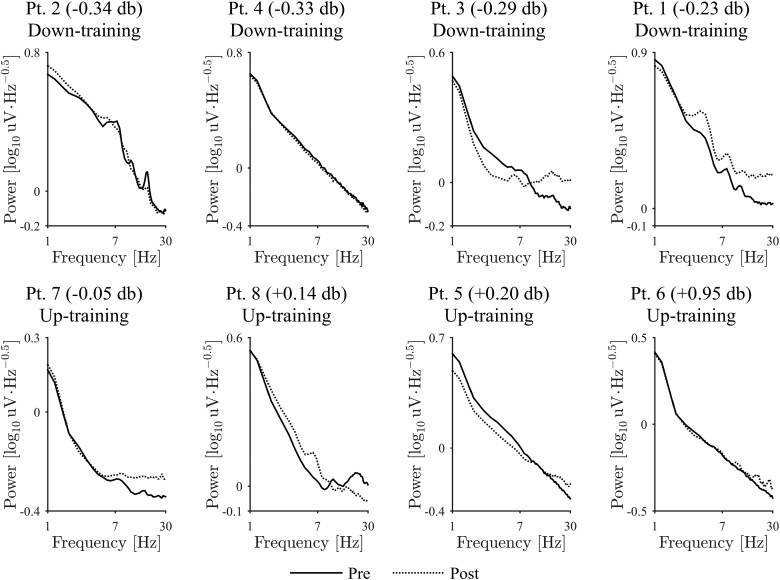
Power spectra of EMG during the pre- and post-feedback sessions. Solid and dashed lines denote the power spectra during resting state before and after the feedback training, respectively. Frequency is shown on a log scale. Each plot shows the patient ID in the title and the difference of β-band power at the selected DBS contacts in the post-feedback session compared to the pre-feedback session. The plots are ordered from left to right, then top panels to bottom panels, so that the differences of β-band power are sorted in ascending order.

We recorded the EMG signals of the forearm contralateral to the selected contacts during the resting state. For Patients 1, 2, and 3, the power spectrum of the EMG demonstrated peaks between 4 and 10 Hz, which corresponded to the tremor (Fig. 6). Although the β-band power changed significantly in the targeted direction, the EMG power between 4 and 10 Hz measured from the contralateral hand to the selected contacts did not change consistently after feedback (*p* = 0.627, two-tailed permutation test; Table 3, c; for EMG change of each patient; Fig. 6).

According to the patients’ reports after feedback training (Table 4), some patients tried to control the radius of the feedback circle through strategies relating to movement intentions. However, we observed no apparent movements or EMG activity caused by movements during the feedback training, and there were no consistent relationships between the β-band power and the EMG power during the training (*p* = 0.466, two-tailed unpaired *t* test; Table 3, d).

**Table 4.** Patients’ reports about feedback training

**Patient ID**	**Patients’ comments after training**
1	I tried to make the circle smaller by narrowing my eyes.
2	(This patient did not report.)
3	Doing something hard, but not to the extent of moving my body, made the circle smaller. I think the circle became small.
4	I was expecting the end of the task. I could not find any control strategy.
5	It seemed that narrowing my eyes made the circle smaller. I think I performed fairly well.
6	Movements of right limbs seemed to make the circle smaller. However, neither moving my eyes nor focusing on an emotion such as happiness or sadness changed the size of the circle.
7	I saw two fixation points. Attempting to merge the points into one made the circle smaller.
8	I have no idea how I could make the circle smaller; but I think the circle became small. I was expecting the end of the task.

## Discussion

The β-band power of STN was demonstrated to be voluntarily modulated through feedback training by patients with Parkinson’s disease. Moreover, the induction of the alteration in the β-band power of STN was not significantly correlated to the motor intention during the training and the EMG power during the resting states.

It should be noted that the β-band power was successfully decreased for all patients in the down-training group and for two of four patients in the up-training group. The patients with Parkinson’s disease may have had difficulty increasing the β-band power during the resting state because the β-band power was already high because of the pathophysiology of the disease.

Although the β-band power during resting state was successfully changed by the feedback training, the patients’ symptoms, especially tremor, had no apparent change. The 10 min feedback training might not be long enough to induce symptomatic alterations. Long-term effects of neurofeedback are expected with more frequent and longer feedback training using the adaptive DBS system that can transmit signals wirelessly. It might also be possible that the β-band power does not cause the tremor symptoms directly. Recent studies suggested that the phase-amplitude coupling between β-phase and high-γ amplitude in the primary motor cortex causes the characteristic tremor of Parkinson’s disease rather than the simple β-band power (de Hemptinne et al., 2013, 2015). Neurofeedback training using the phase-amplitude coupling might improve this symptom. Neurofeedback training with online evaluation of the abnormal oscillation may be used to demonstrate the pathophysiological relationship between the abnormal oscillation and symptoms.

In our experiments, we instructed patients to control the circle without moving their bodies. Patients were unaware that the circle was related to the STN activities that are modulated by movement. However, one patient reported that he tried a strategy relating to limb movement. It is possible that patients involuntarily thought about movements during training, but failed to report these thoughts afterward (either because they forgot or they simply wished to conform to the instructions not to move the body). However, we did not observe any apparent movement during the feedback training, nor did we see a consistent correlation between β-band power in STN and forearm EMG power. Thus, the data indicate that explicit motor intention had little effect on controlling the feedback circle in this training, and our results demonstrate that the neurofeedback system was able to induce a significant alteration in the β-band power during a resting state regardless of the explicit movement intentions.

### Conclusion

Our feedback training successfully demonstrated that the β-band power of the STN could be modulated to increase or decrease based on the patients’ voluntary control. The neurofeedback training may be an effective method for revealing the pathophysiological role of the abnormal oscillations and for developing a novel treatment for Parkinson’s disease.

## Synthesis

Reviewing Editor: Li Li, New York University Shanghai Decisions are customarily a result of the Reviewing Editor and the peer reviewers coming together and discussing their recommendations until a consensus is reached. When revisions are invited, a fact-based synthesis statement explaining their decision and outlining what is needed to prepare a revision will be listed below. The following reviewer(s) agreed to reveal their identity: Tanuj Gulati.

Both reviewers are satisfied with the revision. However, Reviewer 2 has some remaining minor concerns that should be addressed before the ms is accepted for publication. See the details below:

1. When they compare the induced changes in each patient, they consider the power of the beta band in windows of 1sec of time (n = 300), whereas when the group effect is tested only the average power is considered which reduces the variability for the statistical processing.

2. In the manuscript, the validation of the custom software is not reported. It is not shown that software can detect changes in β-band power in other registers as cortical activity in normal people

3. The main comments are regarding the description of the methodology.

In line 103-104 it was written;

“During the 10-minute feedback session, patients were

instructed to make the radius of a black circle on a computer screen smaller by using

their thoughts somehow, without moving their bodies...”

However, there are not detail about the graphic representation of β-band power in particulary , circle size observed by patients, and if the size could be proper for a clear perception of the change by patients.

It is mentioned a normalization of β-band power to scaling circle, but this process is not detailed.

In the manuscript they propose to measure the power of the EMG within the frequency range of 4 to 10 HZ as an index of modification of the motor symptoms, however, in the introduction this is not mentioned as an objective.
